# A silent trio: Giant descending aortic aneurysm combined with coarctation and persistent left brachiocephalic truncus

**DOI:** 10.1002/ccr3.4721

**Published:** 2021-08-25

**Authors:** Maksim Basho, Deniona Nunci, Gentian Vyshka, Edvin Prifti

**Affiliations:** ^1^ Service of Radiology University Hospital Center “Mother Teresa” Tirana Albania; ^2^ Biomedical and Experimental Department Faculty of Medicine University of Medicine in Tirana Tirana Albania; ^3^ Division of Cardiac Surgery University Hospital Center “Mother Teresa” Tirana Albania

**Keywords:** coarctation, descending aorta, giant aneurysm, left brachiocephalic truncus

## Abstract

We report a case of an undiagnosed descending aortic aneurysm, combined with coarctation and persistent left brachiocephalic truncus in a 59‐year‐old male. It highlights the necessity for aortic imaging, when facing a poorly controlled hypertension.

## Graphical abstract

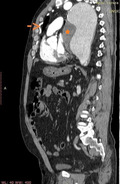

## 1 INTRODUCTION

**FIGURE 1:**
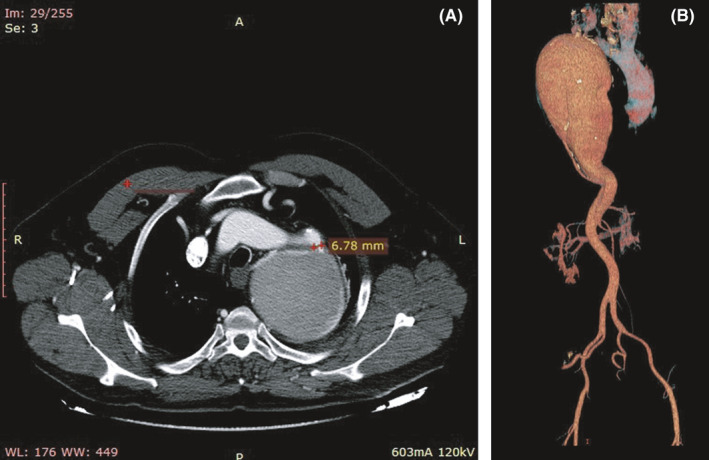
(A) Descending aorta presented extremely dilated, with a transverse diameter approximating ten centimeters and with a coarcted portion proximally with a diameter of less than 7 mm (markers). (B) Angio CT reconstruction images showing the giant aneurysm

A 59‐year‐old Albanian man presented for radiological evaluation following a two‐week period of effort dyspnea. He had a poorly controlled hypertension and was a lifetime heavy smoker (more than twenty cigarettes daily). During a cardiological consultancy, his blood pressure was 170/120 mm Hg, with a heart rate of 110 beats per minute. A diastolic murmur was the only finding in the auscultation, and an electrocardiogram was considered within normality. A CT angiogram of the thorax was performed the same day, with images of a coarcted portion whose diameter was less than 7 millimeters in the axial image, followed from a dilated, giant descending aortic aneurysm that reached a maximum of 9.8 cm in transverse diameter (Figure 1A and B).

**FIGURE 2:**
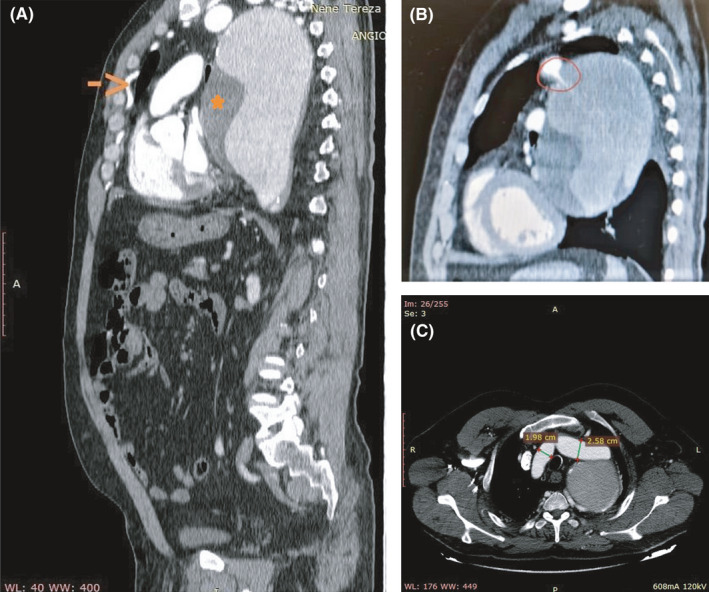
(A) Sagittal thorax reconstructed CT images showing the presence of hypertrophic intercostal arteries (arrow) as well as the giant aneurysm with a dissected flap (asterisk). (B) Coarctation neck (drawn circle) in the sagittal, contrast‐enhanced image. (C) Presence of right and left brachiocephalic truncus, with respective transverse diameters in centimeters

A dissecting flap of more than four centimeters in length was well visualized. Hypertrophic intercostal arteries were present at the sagittal reconstructed CT images (Figure 2A). The coarctation neck was visible in the sagittal reconstructed contrast‐enhanced CT images (Figure 2B). The patient was never diagnosed previously for coarctation; furthermore, the axial CT images showed the presence of left brachiocephalic artery (Figure 2C).

Giant asymptomatic aortic aneurysms are rarely reported, but can still be encountered, presenting an occurrence of major concern in emergency medicine.^1^, ^2^ The presence of hypertrophic intercostal arteries is considered as compensatory and has been reported previously.^3^ Different conditions might contribute to such a complicated clinical picture of a simultaneous coarctation with the huge descending and dissected aortic aneurysm. Some cardiac pathologies may be etiologically important, here including a probable Marfan syndrome, a bicuspid aortic valve, aortic arteritis and syphilis, among other.^4^ In our case, a two‐staged surgical repair was performed, initially with an arch aortic graft; then, the descending aneurysm was repaired via a left thoracotomy at a four‐week distance, a technique originally named as the “elephant trunk” one.^5^

## CONFLICT OF INTEREST

The authors declare no conflict of interest related to this article.

## AUTHOR CONTRIBUTIONS

MB, DN, GV and EP: involved in manuscript writing, data collection, and literature reviewing.

## Data Availability

Data sharing not applicable to this article as no datasets were generated or analysed during the current study.
